# MicroRNA and mRNA Features of Malignant Pleural Mesothelioma and Benign Asbestos-Related Pleural Effusion

**DOI:** 10.1155/2015/635748

**Published:** 2015-02-01

**Authors:** Guntulu Ak, Sandra C. Tomaszek, Farhad Kosari, Muzaffer Metintas, James R. Jett, Selma Metintas, Huseyin Yildirim, Emine Dundar, Jie Dong, Marie Christine Aubry, Dennis A. Wigle, Charles F. Thomas

**Affiliations:** ^1^Department of Chest Diseases, Medical Faculty, Eskisehir Osmangazi University, 26480 Eskisehir, Turkey; ^2^Division of Thoracic Surgery, Department of Surgery, Mayo Clinic, Rochester, MN 55905, USA; ^3^Department of Molecular Medicine, Mayo Clinic, Rochester, MN 55905, USA; ^4^Division of Oncology, National Jewish Health, Denver, CO 80206, USA; ^5^Department of Public Health, Medical Faculty, Eskisehir Osmangazi University, 26480 Eskisehir, Turkey; ^6^Department of Pathology, Medical Faculty, Eskisehir Osmangazi University, 26480 Eskisehir, Turkey; ^7^Department of Laboratory Medicine and Pathology, Mayo Clinic, Rochester, MN 55905, USA; ^8^Division of Pulmonary & Critical Care Medicine, Mayo Clinic, Rochester, MN 55905, USA

## Abstract

*Introduction*. We investigated the expression of microRNAs and mRNAs in pleural tissues from patients with either malignant pleural mesothelioma or benign asbestos-related pleural effusion. *Methods*. Fresh frozen tissues from a total of 18 malignant pleural mesothelioma and 6 benign asbestos-related pleural effusion patients were studied. Expression profiling of mRNA and microRNA was performed using standard protocols. *Results*. We discovered significant upregulation of multiple microRNAs in malignant pleural mesothelioma compared to benign asbestos-related pleural effusion. Hsa-miR-484, hsa-miR-320, hsa-let-7a, and hsa-miR-125a-5p were able to discriminate malignant from benign disease. Dynamically regulated mRNAs were also identified. MET was the most highly overexpressed gene in malignant pleural mesothelioma compared to benign asbestos-related pleural effusion. Integrated analyses examining microRNA-mRNA interactions suggested multiple altered targets within the Notch signaling pathway. *Conclusions*. Specific microRNAs and mRNAs may have diagnostic utility in differentiating patients with malignant pleural mesothelioma from benign asbestos-related pleural effusion. These studies may be particularly helpful in patients who reside in a region with a high incidence of mesothelioma.

## 1. Introduction

Malignant pleural mesothelioma (MPM) is an aggressive tumor and remains as a significant worldwide health problem because of its poor prognosis and increasing incidence [1]. The major known risk factor is exposure to asbestos. Although MPM has a poor clinical outcome and is frequently untreatable, recent series have demonstrated that early diagnosis and aggressive treatment strategies may improve overall survival.

The detection of early stage patients with MPM and the differentiation of early stage MPM from benign asbestos-related pleural effusion (BAPE) are critically important for improving the survival of MPM patients. The typical histologic findings of BAPE on pleural tissue are nonspecific pleuritis/fibrosis, which is a nondiagnostic entity. The diagnosis of BAPE is based on a history of asbestos exposure and exclusion of other causes, together with a period of observation to exclude malignancy [2]. Unfortunately, there is no reliable clinical, radiological, or laboratory data that can reliably differentiate early stage MPM from BAPE.

Recent advances in cancer molecular biology include the identification of microRNAs (miR) involved in regulating gene expression at the posttranscriptional level. It has been shown that approximately half of the known miR are located in cancer-associated genomic regions or in fragile sites [3]. They can act as oncogenes or tumor suppressors [4–6]. Studies have shown that miR profiling can differentiate tumor from normal cells, different tumor histological subtypes from each other, and may potentially predict clinical outcome [6–8]. miR have multitarget characteristics and can regulate groups of genes; thus, low-level modulation from individual miR could have an additive effect on multiple gene targets [9].

One can speculate that the analysis of expression signatures for miR and mRNAs in patients with MPM could differentiate benign pleural disease or metastatic cancer to the pleura from MPM, be useful for all of the histological subtypes of MPM, correlate with the extent of disease in order to monitor treatment response, and predict outcome. Only a small number of studies have been done in MPM to examine miR expression [10–19] and corresponding gene expression of these tumors [20–25].

In this study, we investigated the miR and mRNA expression levels and their integrated analysis in both BAPE and different stages and histological subtypes of MPM.

## 2. Materials and Methods

### 2.1. Patients and Specimens

A total 18 MPM and 6 BAPE patients who were diagnosed and followed at the Eskisehir Osmangazi University Hospital in Turkey were enrolled in this study. None of the patients had received chemotherapy or radiotherapy prior to diagnosis. Clinical data including age, sex, asbestos exposure history, histology, stage, treatment history, and survival characteristics were collected from all mesothelioma patients, while age, sex, asbestos exposure history, and survival data were collected from the BAPE patients (Table 1). Follow-up was conducted for three years or until death. The study was approved by the Local Ethics Committee in Eskisehir Osmangazi University, and all of the patients provided written informed consent for genetic analysis on their pleural specimens.

Pleural specimens were obtained by medical thoracoscopy, CT-guided Abrams needle, or thoracotomy. Biopsy specimens were processed with a portion frozen for future analysis and another part fixed in formalin and sent to the pathology department for diagnosis. All biopsy samples underwent review in Turkey by a single pathologist and were subsequently confirmed by a lung pathologist at Mayo Clinic. Histological diagnosis of MPM was confirmed using immunohistochemistry. Diagnosis of BAPE was based on a nonmalignant pleural biopsy, a history of asbestos exposure, and the exclusion of the other causes, together with a three-year observation period to exclude malignancy. The frozen samples were transported to the Mayo Clinic for gene expression and miR analysis. Mesothelioma specimens with a tumor cellular content greater than 50% of the biopsy were used for experiments.

### 2.2. RNA Isolation

Total RNA was extracted from macrodissected fresh-frozen samples using the RNeasy Mini Kit (Qiagen, Valencia, CA) according to the manufacturer's instructions. Total RNA quantity was checked by NanoDrop ND-1000 Spectrophotometer (Thermo Fisher Scientific Inc., Wilmington, DE). Adequate RNA quality was confirmed by the Agilent 2100 Bioanalyzer (Agilent Technologies Inc., Santa Clara, CA).

### 2.3. Gene Expression Profiling

The mRNA expression profiling was performed using the Affymetrix GeneChip Human Genome U133 Plus 2.0 array analyzing the expression level of 47,000 transcripts including over 20,000 known human genes (Affymetrix, Santa Clara, CA). Microarray experiments were conducted by the Mayo Clinic Advanced Genomic Technology Center Microarray Shared Resource using the Affymetrix One Cycle Target Labeling and Control Reagents kit (Affymetrix, Santa Clara, CA). Briefly, 3–5 *µ*g of total RNA was used to synthesize double-stranded complementary DNA (cDNA) using SuperScript II reverse transcriptase (Invitrogen, Carlsbad, CA) and T7 Oligo (dT) primers. Subsequently, the products were column purified (Affymetrix, Santa Clara, CA) and then in vitro transcribed to generate biotin-labeled cRNA. The IVT products were then column purified, fragmented, and hybridized onto Affymetrix U133 Plus 2.0 GeneChips at 45°C for 16 h. Following hybridization, the arrays were washed and stained with streptavidin-phytoerythrin and then scanned in an Affymetrix GeneChip Scanner 3000 (Affymetrix, Santa Clara, CA). All control parameters were confirmed to be within normal ranges before normalization and data reduction was initiated.

### 2.4. MicroRNA Profiling

Total RNA was reverse transcribed into cDNA using the TaqMan MicroRNA Reverse Transcription Kit and the Megaplex RT Primers, Human Pool A (Applied Biosystems, Foster City, CA). MicroRNA expression profiling was performed using the TaqMan Universal PCR Master Mix. For microRNA expression profiling, per sample 6 *μ*L of Megaplex RT product was combined with 450 *μ*L of TaqMan Universal PCR Master Mix, No AmpErase UNG (Applied Biosystems, Foster City, CA) and 444 *μ*L of nuclease free water. After brief centrifugation, 100 *μ*L of the PCR Reaction Mix was loaded into each port of the TaqMan Human MicroRNA Array Card A (Applied Biosystems, Foster City, CA). The array was centrifuged at 1200 rpm for 1 min in a Sorvall Legend RT centrifuge (Kendro Laboratory Products, Newtown, CT) and sealed before running. The arrays were run on the 7900HT fast Real-Time PCR System (Applied Biosystems, Foster City, CA) according to the manufacturer's instructions. The SDS v2.3 software was set up for a 384-well TaqMan Low Density Array with Relative Quantification (ΔΔ*C*_*T*_).

### 2.5. Data Analysis

#### 2.5.1. MicroRNA Data

MPM and BAPE miR data were normalized separately to a miR mammalian U6 endogenous control gene, utilizing the RQ Manager software. Cycle threshold (*C*_*T*_) values greater than 35 were set to 35. The average Δ*C*_*T*_ values were calculated by subtracting the average endogenous control *C*_*T*_ value from the average miR *C*_*T*_ value. The standard deviation of the difference was calculated from the standard deviation of the miR and endogenous control values. The results of Δ*C*_*T*_ were mean ± S.D. Low miR Δ*C*_*T*_ value corresponded to high miR expression. The calculation of ΔΔ*C*_*T*_ involved subtraction by the Δ*C*_*T*_ calibrator value. This was subtraction of one of the Δ*C*_*T*_ values as an arbitrary constant. The standard deviation of ΔΔ*C*_*T*_ was the same as the standard deviation of the Δ*C*_*T*_ value. The fold changes for relative miR expression were determined by 2^−Δ(Δ*C*_*T*_)^. miR which did not detect any of samples were excluded from analyses.

Student's *t*-test was used to compare the expression of each miR between MPM and BAPE. All corrections for multiple hypothesis testing (miR and Affymetrix) were by *q* value function in R [26]. Only upregulated miR passed criteria for multiple hypothesis testing (*q*-value < 0.05). miR with more than 3 mesothelioma samples with *C*_*T*_ > 35 were not reported as upregulated (Table 2).

Receiver operating characteristics (ROC) curves were plotted and the area under the curve (AUC) computed to access the individual ability of each miR to differentiate BAPE from MPM and on the histology and stage of MPM.

#### 2.5.2. Gene Expression Data

The microarray signal intensity (.CEL) files were normalized and processed by the “gcrma” package in R (http://www.bioconductor.org/) to calculate the Log_2_ intensity values for each probeset. To identify the most differentially expressed genes in MPM versus BAPE, probesets were ranked by signal to noise ratio calculated as SNR = (*m*_MPM_ − *m*_BAPE_)/(*s*_MPM_ + *s*_BAPE_) where *m*'s were mean expression values and *s*'s were maximum of 0.4 × *m* and standard deviation [27]. SNR values greater than and less than zero potentially indicate over- and underexpression in MPM compared with BAPE, respectively. We also required that the average expression in samples overexpressing a gene had greater than 3.5 Log_2_ intensities. Log_2_ expression intensities for the gcrma normalized data ranged from 2 to 15. Based on our experience with quantitative RT-PCR, gene expression intensities below 3.5 were not reliable and frequently not detected. Significant figures for overexpression in MPM compared with BAPE were calculated by *t*-test and then corrected for multiple comparison correction using the “*q* value” package in R [26]. Downregulated probesets in MPM compared with BAPE did not pass our criteria for multiple hypothesis testing (*q*-value < 0.05). Upregulated probesets with best SNR and *q* < 0.01 were reported.

#### 2.5.3. miRNA and mRNA Integrated Analysis

We used the mgsa program in R for integrated analysis to identify canonical pathways altered in MPM [28]. Upregulated probesets (2270) with SNR > 0.2 and *P* value < 0.01 were selected. These probesets represented 1740 unique genes. Also, gene targets of 5 miR with significant downregulation in MPM (*P* < 0.05) were identified using the c3 data set in version 3.1 of the molecular signature database (MSDB) from the Broad Institute (http://www.broadinstitute.org/gsea/msigdb/index.jsp). These targets included 1258 unique genes. The list of upregulated genes and targets of downregulated miR were combined and used in the canonical pathway analysis using c2.cp in MSDB (version 3.1). Reported results were based on 1275 genes in the combined list that mapped to c2.cp.

## 3. Results

Eighteen MPM and 6 BAPE patients were included in this study. The mean age was 68.0 ± 7.5 years for MPM patients and 65.7 ± 12.3 years for BAPE patients. In our MPM group, the male-to-female ratio was equal while it was 5 : 1 in the BAPE group. Most of the MPM patients had epithelial type histology and advanced stage disease. Of these only one patient received multimodal treatment, while 10 received chemotherapy and 7 had best supportive care (Table 1).

**Figure 1:**
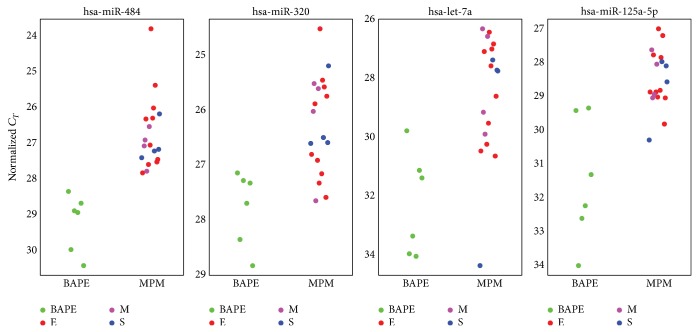
Representative overexpressed microRNAs.

We discovered differential expression of miR between the MPM and BAPE samples. Eleven miR were significantly upregulated in MPM compared to BAPE and included hsa-miR-484 (fold change 5.58), hsa-miR-320 (fold change 2.87), hsa-let-7a (fold change 13.93), hsa-miR-744 (fold change 4.26), hsa-miR-20a (fold change 5.7), hsa-miR-193b (fold change 3.03), hsa-let-7d (fold change 5.82), hsa-miR-125a-5p (fold change 8.17), hsa-miR-92a (fold change 2.39), hsa-miR-155 (fold change 3.16), and hsa-miR-152 (fold change 2.93) (Table 2, Figure 1).

We then evaluated the diagnostic value of individual miR to differentiate MPM from BAPE using ROC analysis and AUC for all significant miR. Four of 11 miR, hsa-miR-484, hsa-miR-320, hsa-let-7a and hsa-miR-125a-5p had ≥0.90 of AUC values: hsa-miR-484 with ≤8.15 cut-off value of Δ*C*_*T*_ had 100% sensitivity and specificity to discriminate MPM from BAPE, while cut-off values of Δ*C*_*T*_, sensitivity, and specificity for hsa-miR-320, hsa-let-7a, and hsa-miR-125a-5p were ≤7.27 versus 78% and 100%; ≤11 versus 94% and 83%; ≤9.36 versus 89% and 100%, respectively.

Within the MPM samples, there did not appear to be any significant miR expression differences among epithelial, sarcomatous, and mixed type histology or significant differences between early stage (I-II) and late stage (III-IV) malignant disease.

**Figure 2:**
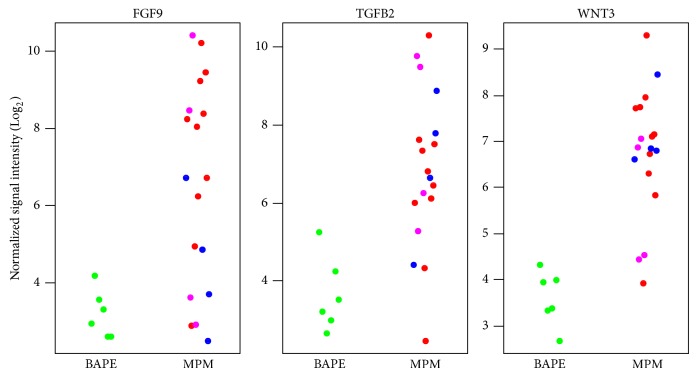
Representative overexpressed mRNAs.

Microarray analyses were performed to identify differentially expressed mRNA. We identified a number of dynamically regulated mRNAs including members from the FGF (FGF9), TGFB (TGFB2), and WNT (WNT3) signaling pathways (Table 3, Figure 2).

We also identified multiple mRNA targets of miR. From integrated analysis we identified eight significant pathways, including two pathways related to NOTCH signaling (Table 4).

**Figure 3:**
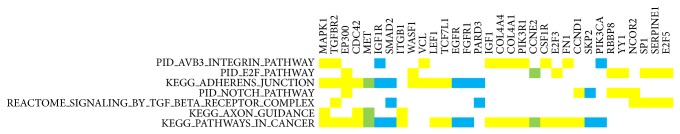
Selected pathways and genes by an integrated analysis using overexpressed mRNA (microarray) and targets genes of underexpressed miR. Yellow depicts targets of underexpressed miR and blue depicts overexpressed genes by microarray. Green depicts overexpressed genes that are also targets of underexpressed miR. There were 232 genes in the 8 pathways. Only 32 genes that participated in more than 1 pathway are shown. Only one of two pathways related to NOTCH signaling was included in creating this figure. MAPK1, TGFBR2, EP300, CDC42, MET, IGF1R, and SMAD2 participate in 3 or more pathways. MET and CCNE2 mRNAs are overexpressed and are targets of underexpressed miR.

Genes related to these pathways are described in Supplementary Table 1. (see Supplementary Materials available online at http://dx.doi.org/10.1155/2014/635748).  Figure 3 describes 32 genes that participated in more than 1 of these pathways. MAPK1, TGFBR2, EP300, CDC42, MET, IGF1R, and SMAD2 participated in three or more pathways, suggesting potential targets for therapy (Figure 3).

Interestingly, MET and CCNE2 were overexpressed in MPM and were also targets of underexpressed miR. Other than these two, there were 13 other overexpressed genes in MPM that were also targets of underexpressed miR. Importantly, MET was the most highly overexpressed gene with over 15-fold overexpression in MPM compared to BAPE (Table 5). This finding is consistent with the literature regarding the role of the c-MET oncogene in mesothelioma.

## 4. Discussion

Differentiating between MPM and BAPE on pleural biopsy can be difficult in some cases, even with the use of immunohistochemistry on biopsy samples obtained by thoracoscopy. When the histological finding of pleural tissue is called nonspecific pleuritis/fibrosis, there are no definitive clinical, radiological, or laboratory data that can help clinicians determine the next step in management. One is therefore left to consider one of two approaches: observation with prolonged follow-up or further biopsies. Both of these considerations are not ideal, especially for individuals residing in regions where they are exposed to environmental asbestos and the incidence rate of mesothelioma is high. Patients with nonspecific pleuritis/fibrosis on thoracoscopic biopsy can be diagnosed as MPM within one year [29]. MPM patients who have early stage disease can be candidates for aggressive treatment and may have improved survival if they are diagnosed at the presentation and not during prolonged observation.

Several studies have evaluated the expression levels of miR in MPM [10–19]. Some of these studies reported miR expression levels comparing MPM and normal samples [10–12, 15–19] while others reported miR expression levels in MPM and other cancers including lung adenocarcinoma [13, 14]. None to date have examined miR expression levels to differentiate MPM from BAPE. In this study, we examined the miR and mRNA expression levels and their integrated analysis in both MPM and BAPE.

Guled and colleagues showed that in MPM compared with normal samples, twelve miR were highly expressed whereas nine other miR either were not expressed or had severely reduced expression levels [11]. In another study of MPM and human normal mesothelial cell cultures, 22 differentially expressed miR were identified, some of which have been linked to oncogenesis as members of the miR-17-92 cluster [15].

Benjamin and colleagues developed an assay which differentiates MPM from carcinomas using miR expression [13]. They found that the hsa-miR-200 family is strongly expressed in adenocarcinoma samples from a variety of epithelial tissues but minimally so in MPM [13]. Gee and colleagues identified a panel of miR that were specifically downregulated in MPM (irregardless of histological subtype) compared to lung adenocarcinoma and included miR-141, miR-200a^*^, miR-200b, miR-200c, miR-203, miR-205, and miR-429 [14]. They also evaluated the individual ability of these miR to distinguish between sample types and found that all were good discriminators of disease. The authors concluded that when the pathological tests are inconclusive, measuring a combination of miR could lead to an accurate diagnosis [14].

Several studies have evaluated miR as a new potential biomarker for the diagnosis of mesothelioma comparing the expression levels of miR in mesothelioma and asbestos exposed control groups using ROC analysis [17–19]. Santarelli et al. found that miR-126 could significantly differentiate high-risk individuals from mesothelioma patients with a sensitivity of 73% and specificity of 74% [17]. Another study reported that cut-off values for miR-103 could discriminate mesothelioma patients from asbestos exposed controls, revealing a sensitivity of 83% and specificity of 71% [18]. Further studies demonstrated that miR-625-3p level was significantly elevated in serum from mesothelioma patients compared with asbestosis patients with a sensitivity of 70% and a specificity of 90% [19].

We discovered a number of miR that were significantly upregulated in MPM compared to BAPE. When we evaluated the diagnostic value of individual miR to differentiate MPM from BAPE, hsa-miR-484, hsa-miR-320, hsa-let-7a, and hsa-miR-125a-5p had ≥0.90 AUC values. Therefore, we believe that hsa-miR-484, hsa-miR-320, hsa-let-7a, and hsa-miR-125a-5p could play an important role in discriminating MPM from BAPE.

Previous studies have reported different expression levels of several miR among histopathological subtypes of MPM [11, 12]. Among our samples we did not find differentially expressed miR related to histopathological subtypes, likely because the majority of our samples were epithelial. In regard to the stage of MPM, results have been contradictory, possibility related to small sample sizes. For example, some authors found no correlations with miR expression and tumor stage, while other studies showed that some miR were downregulated in advanced stage MPM [12, 17].

We also identified several genes with altered expression from the FGF (FGF9), TGFB (TGFB2), and WNT (WNT3) signaling pathways. Gee et al. used target prediction software to identify proteins predicted to be downregulated by miR [14]. They then queried their results with two different predictive algorithms to find targets with a higher probability of interactions. They predicted that the downregulation of unique miR would result in the loss of multiple levels of posttranscriptional gene regulation of the WNT signaling pathways [14].

Specific growth factors such as epidermal growth factor (EGF), hepatocyte growth factor (HGF), vascular endothelial growth factor (VEGF), and insulin-like growth factor (IGF) and their receptors have been shown to play a significant role in the oncogenesis, progression, and resistance to therapy in mesothelioma [30]. HGF is a multifunctional growth factor that can induce many important biological functions related to the malignant phenotype, including cell scattering, invasion, proliferation, and morphogenesis. HGF induces these biological functions through binding to its transmembrane tyrosine kinase receptor, c-mesenchymal-epithelial transition (c-MET) [31]. The majority of mesothelioma cases express c-MET [32]. We performed an integrated analysis of mRNA and miR interactions and also found overexpression of c-Met mRNA and corresponding underexpression of its targeted miR (Table 5). Our data suggest that the c-MET pathway may have promise as a therapeutic target in future clinical trials.

HGF and c-Met are highly expressed in SV40-positive mesothelioma samples [33–35]. It has been shown that SV40 induces HGF/Met receptor activation, telomerase activity, and Notch-1 activation in human mesothelial cells and MPM biopsy samples [33–35]. When SV40 infects human mesothelial cells it causes Met activation via an autocrine loop. Furthermore, SV40 replicates in human mesothelial cells and infects adjacent human mesothelial cells, inducing an HGF-dependent Met activation and cell cycle progression into S phase [33]. Notch signaling pathways were found in our study but we do not have data regarding the SV40 status in our patient population.

## 5. Conclusions

We have identified specific miR as having potential diagnostic utility in patients with MPM or BAPE. We believe that evaluation of miR would be helpful in asbestos exposed patients diagnosed with nonspecific pleuritis/fibrosis on a pleural biopsy, especially if a patient comes from a region with high mesothelioma incidence rates. Also, we have identified specific genes and signaling pathways that may have promise as a therapeutic target in patients with MPM. These results will need to be validated in a larger cohort of patients to confirm their diagnostic and therapeutic utility.

## Supplementary Material

Selected pathways by an integrated analyses of mRNA and miRNA data. Over-expressed genes and targets of under-expressed miRNA in mesothelioma are shown in blue and yellow shading, respectively. Genes in green shading were over-expressed and also targets of under-expressed miRNA. These included a number of prominent cancer genes such as MET and CCNE2.

## Figures and Tables

**Table 1:** Patient demographics.

	MPM (*n* = 18)	BAPE (*n* = 6)
Age, y ± SD (range)	68.0 ± 7.5 (48–81)	65.7 ± 12.3 (49–79)
Sex, male : female	9 : 9	5 : 1
Asbestos exposure time,	33.1 ± 19.6 (0–81)	28.2 ± 11.3 (20–49)
y ± SD (range)		
Histology, *n* (%)		
Epithelial	10 (55.6)	—
Mixed	4 (22.2)	
Sarcomatoid	4 (22.2)	
Stage, *n* (%)		
I-II	4 (22.2)	—
III-IV	14 (77.8)	
Treatment, *n* (%)		
Yes	11 (61.1)	—
No	7 (38.9)	

MPM: malignant pleural mesothelioma; BAPE: benign asbestos-related pleural effusion; SD: standard deviation.

**Table 2:** Average Δ*C*_*T*_ value of overexpressed microRNAs in malignant pleural mesothelioma compared with benign asbestos-related pleural effusion.

	MPM	BAPE	Fold change	*q* value^*^
hsa-miR-484	7.06	9.54	5.58	0.010
hsa-miR-320	6.62	8.14	2.87	0.017
hsa-let-7a	8.85	12.65	13.93	0.019
hsa-miR-744	11.67	13.76	4.26	0.019
hsa-miR-20a	10.65	13.16	5.7	0.019
hsa-miR-193b	8.09	9.69	3.03	0.019
hsa-let-7d	10.18	12.72	5.82	0.045
hsa-miR-125a-5p	8.8	11.83	8.17	0.045
hsa-miR-92a	9.93	11.19	2.39	0.045
has-miR-155	8.77	10.43	3.16	0.045
hsa-miR-152	11.93	13.48	2.93	0.047

^*^[26]. MPM: malignant pleural mesothelioma; BAPE: benign asbestos-related pleural effusion.

**Table 3:** mRNAs overexpressed in malignant pleural mesothelioma compared with benign asbestos-related pleural effusion.

Probeset	Gene symbol	^*^ *q*-greater	SNR	BAPE	MPM	FC
239178_at	NA	<0.01	0.91	3	6.5	11.5
216074_x_at	WWC1	<0.01	0.9	3	6.3	10.2
205074_at	SLC22A5	<0.01	0.84	3.3	6.6	9.9
206404_at	FGF9	<0.01	0.84	3.2	6.5	10
200637_s_at	PTPRF	<0.01	0.82	3.7	7.3	12
226799_at	FGD6	<0.01	0.8	3.2	6.2	8
213085_s_at	WWC1	<0.01	0.8	3.7	7.1	10.9
228121_at	TGFB2	<0.01	0.76	3.7	6.9	9.2
227769_at	NA	<0.01	0.76	3.6	6.8	8.8
211029_x_at	FGF18	<0.01	0.76	3.1	5.8	6.6
229103_at	WNT3	<0.01	0.76	3.6	6.8	8.8
231382_at	FGF18	<0.01	0.73	3.6	6.6	7.9
226591_at	NA	<0.01	0.73	3.2	5.8	6.1
212325_at	LIMCH1	<0.01	0.71	4.4	7.9	11.1
228523_at	NANOS1	<0.01	0.7	3.3	5.8	5.7
205729_at	OSMR	<0.01	0.68	2.9	5.2	4.6
204519_s_at	PLLP	<0.01	0.68	4.6	8	10.5
209631_s_at	GPR37	<0.01	0.66	3.2	5.5	4.9

^*^[26]. Used GCRMA in the R package for normalization of microarray data.

SNR: signal to noise ratio; BAPE: benign asbestos-related pleural effusion; MPM: malignant pleural mesothelioma; FC: fold change.

**Table 4:** Integrated analysis of mRNA and microRNAs.

	In population	In study set	Estimate	^*^mRNA	^**^miRNA	^***^Both
KEGG_AXON_GUIDANCE	129	38	0.94	12	28	2
PID_NOTCH_PATHWAY	59	23	0.61	6	17	0
REACTOME_SIGNALING_BY_TGF_BETA_RECEPTOR_COMPLEX	63	23	0.56	10	14	1
KEGG_ADHERENS_JUNCTION	75	28	0.54	15	14	1
PID_AVB3_INTEGRIN_PATHWAY	75	34	0.47	8	27	1
REACTOME_SIGNALING_BY_NOTCH	103	30	0.42	16	18	4
PID_E2F_PATHWAY	74	24	0.41	11	16	3
KEGG_PATHWAYS_IN_CANCER	328	88	0.40	45	49	6

^*^Number of overexpressed mRNAs in the pathway.

^**^Number of gene targets of underexpressed microRNAs in the pathway.

^***^Number of common genes in ∗ and ∗∗.

In Study Set: number of genes in the pathway from genes selected for the analysis.

In Population: number of genes in the pathway.

The column “estimate” for MGSA indicates the marginal value of a term being in the “active” state; 0.4 was used as the threshold.

**Table 5:** Overexpressed genes that are also targeted by downregulated microRNAs in pathways from Table 4.

	miRNA data				mRNA data		
	miR-19a *P* = 0.021	miR-29c *P* = 0.027	miR-449a *P* = 0.014	miR-511 *P* = 0.011	Probeset	P-MPM > BAPE	Fold change
MET			X		203510_at	0.0067	15.56
WNT3	X				229103_at	0	8.75
HDAC4	X	X			204225_at	0.0071	4.92
CDK6		X	X		224848_at	0.0069	4.26
RXRA	X				202449_s_at	0.0015	3.51
TBL1XR1			X		222634_s_at	0.0076	2.87
EIF2C2				X	213310_at	2.00*E* − 04	2.83
MTMR4		X			212277_at	0.0089	2.81
CCNE2			X		205034_at	0.0036	2.48
COL12A1			X		231879_at	0.0093	2.45
SMARCA2	X				212257_s_at	0.0099	2.36
EIF2C1	X	X		X	228120_at	0.002	1.93
SEMA4F			X		228660_x_at	1.00*E* − 04	1.82
RAF1	X				1557675_at	0.0077	1.77
TFDP2			X		244043_at	0.0093	1.47

*P* values in microRNAs columns are for MPM < BAPE.

MPM: malignant pleural mesothelioma; BAPE: benign asbestos-related pleural effusion.
